# On the Rate of Synthesis of Individual Proteins within and between Different Striated Muscles of the Rat

**DOI:** 10.3390/proteomes4010012

**Published:** 2016-03-15

**Authors:** Stuart Hesketh, Kanchana Srisawat, Hazel Sutherland, Jonathan Jarvis, Jatin Burniston

**Affiliations:** Research Institute for Sport and Exercise Sciences, Liverpool John Moores University, Liverpool, L3 3AF, UK; S.J.Hesketh@2014.ljmu.ac.uk (S.H.); K.Srisawat@2015.ljmu.ac.uk (K.S.); H.Sutherland@ljmu.ac.uk (H.S.); J.C.Jarvis@ljmu.ac.uk (J.J.)

**Keywords:** deuterium oxide, stable isotope labelling, mass isotopomer distribution analysis, matrix-assisted laser desorption ionisation mass spectrometry, 2D gel electrophoresis, skeletal muscle, cardiac muscle, protein synthesis

## Abstract

The turnover of muscle protein is responsive to different (patho)-physiological conditions but little is known about the rate of synthesis at the level of individual proteins or whether this varies between different muscles. We investigated the synthesis rate of eight proteins (actin, albumin, ATP synthase alpha, beta enolase, creatine kinase, myosin essential light chain, myosin regulatory light chain and tropomyosin) in the extensor digitorum longus, diaphragm, heart and soleus of male Wistar rats (352 ± 30 g body weight). Animals were assigned to four groups (*n* = 3, in each), including a control and groups that received deuterium oxide (^2^H_2_O) for 4 days, 7 days or 14 days. Deuterium labelling was initiated by an intraperitoneal injection of 10 μL/g body weight of 99.9% ^2^H_2_O-saline, and was maintained by administration of 5% (*v*/*v*) ^2^H_2_O in drinking water provided *ad libitum*. Homogenates of the isolated muscles were analysed by 2-dimensional gel electrophoresis and matrix-assisted laser desorption ionisation time of flight mass spectrometry. Proteins were identified against the SwissProt database using peptide mass fingerprinting. For each of the eight proteins investigated, the molar percent enrichment (MPE) of ^2^H and rate constant (*k*) of protein synthesis was calculated from the mass isotopomer distribution of peptides based on the amino acid sequence and predicted number of exchangeable C–H bonds. The average MPE (2.14% ± 0.2%) was as expected and was consistent across muscles harvested at different times (*i.e.*, steady state enrichment was achieved). The synthesis rate of individual proteins differed markedly within each muscle and the rank-order of synthesis rates differed among the muscles studied. After 14 days the fraction of albumin synthesised (23% ± 5%) was significantly (*p* < 0.05) greater than for other muscle proteins. These data represent the first attempt to study the synthesis rates of individual proteins across a number of different striated muscles.

## 1. Introduction

Skeletal muscle accounts for ~40% of adult body mass and, in addition to its obvious function in movement, muscle has important roles in glucose homeostasis and protein metabolism. For example, in healthy individuals muscle is responsible for >75% of insulin-mediated glucose disposal and ~60% of total body protein turnover. Therefore, maintaining adequate muscle mass and function are important factors that impact long-term health. Age-related losses in muscle mass lead to frailty and loss of independence, and so directly impact an individual’s quality of life. Moreover, those individuals with the greatest level of muscle loss are more likely to be obese or suffer from metabolic disorders including impaired glucose tolerance [1]. Furthermore, because skeletal muscle is the major repository of protein/amino acids that can be mobilised during starvation or injury, muscle loss is also an independent predictor of mortality in cachexia associated with diseases such as cancer and chronic heart failure [2].

Muscle mass is determined by the historical balance between the synthesis and degradation of its constituent proteins. Synthesis of new protein *in vivo* has traditionally been investigated by metabolic labelling using tracers such as the stable isotope labelled amino acid l-[*ring*-^13^C_6_]-phenylalanine e.g., [3]. This enables the fraction of newly synthesised protein to be calculated from the precursor: product ratio. Incorporation of stable-isotope labelled amino acids in to protein *in vivo* is most commonly combined with gas-chromatography mass spectrometry (GC-MS) analysis of hydrolysed amino acids to measure the average rate of synthesis in protein mixtures extracted from skeletal muscle of humans and non-human animal models [4]. In rats, the average half-life of proteins is approximately 14 days in mixed-fibre locomotive muscles, whereas in slow-twitch postural muscles protein half-life is estimated to be 7 days [5]. These values represent averages across the entire proteome and so are a gross simplification of events at an individual protein level. Indeed the average turnover rate may be misleading because turnover of the mitochondrial protein, cytochrome c, is more rapid in fast- than slow-twitch muscle [6].

Protein synthesis and degradation are each intricate processes that are regulated independently and on a protein by protein basis to maintain homeostasis or to facilitate muscle adaptation. A greater understanding of synthesis rates at the individual protein level could significantly advance knowledge regarding differences in muscle phenotype and the adaptation of muscle to physiological and pathophysiological stimuli. Watt *et al.* [7] reports differences between fast-twitch muscle extensor digitorum longus (EDL) and slow-twitch soleus in rats during a 2-week regimen of high-intensity jump training. The growth rate of both EDL and soleus increased up to 70% when compared to control, but growth of the fast-twitch muscle was primarily achieved through greater (28%) protein synthesis, whereas in the soleus muscle protein synthesis did not change but there was a 38% decrease in protein degradation. While it is likely some proteins may not have followed these overall trends, until recently it has not been possible routinely to measure isotope incorporation (*i.e.*, synthesis) in large numbers of individual proteins. However, the application of proteomic separation techniques in striated muscle [8] along with advances in the sensitivity of mass spectrometers now enable such work to be undertaken. For example, Jaleel *et al*. [9] reports metabolic labelling with l-[*ring*-^13^C_6_]-phenylalanine *in vivo* and analysis of muscle mitochondria using 2-dimensional gel electrophoresis (2DGE). Gel spots were identified by peptide mass spectrometry, whereas incorporation of the stable isotope label was measured in mixtures of hydrolysed amino acids using GC-MS. The marriage of these established techniques enabled synthesis rates to be calculated for 68 mitochondrial proteins in rat skeletal muscle. Jaleel *et al*. confirms the synthesis of proteins within a muscle differs on a protein by protein basis. Of the proteins investigated beta enolase had the greatest synthesis rate (11%/day) while myosin light chain regulatory had the lowest (3%/day), indicating an approximate 10-fold variation in protein synthesis rates across the proteins investigated. 

A major shortcoming of amino acid tracer studies is that it is impractical to measure the true enrichment of the precursor pool (*i.e.*, aminoacyl-tRNA) directly. This combined with uncertainties regarding the transport of amino acids between intracellular and extracellular compartments, and recycling of amino acids from protein degradation, gives rise to uncertainty regarding the calculation of the renewal rate of the protein products. Secondly, amino acid tracers require intravenous infusion, which is invasive and therefore necessarily restricts the duration of such investigations. As a consequence, it is likely the average synthesis rates calculated from mixtures of muscle proteins are skewed toward that of the more rapidly synthesised proteins. Moreover, linear extrapolation of short-term (e.g., 30 min) incorporation of an amino acid tracer e.g., [10] to longer time periods (e.g.,%/day) will further lead to overestimation because incorporation of labelled amino acid in to the protein product exhibits first-order (rise-to-plateau) kinetics. These shortcomings can be entirely overcome through the use of deuterium oxide (^2^H_2_O or “D_2_O”) to label macromolecular precursors, including amino acids, *in vivo* [11].

^2^H_2_O has the fundamental advantage that it can be administered via drinking water and labelling can be conducted in free-living animals over experimental periods spanning days or months [12]. ^2^H_2_O equilibrates within the body water compartment rapidly (<30 min; in rodents) and H–C bonds of amino acids (principally alanine) become ^2^H-labelled intracellularly via transamination and/or de novo synthesis [13]. Therefore, unlike amino acid tracers, enrichment of the precursor pool is not affected by amino acid metabolism, membrane transport or dilution effects from protein degradation. After incorporation in to newly synthesised protein the ^2^H-label is irreversible [14] and can be quantified by GC-MS of amino acid hydrolysates (e.g., [14]) or by peptide mass spectrometry of tryptic digests. For example, Xiao *et al*. [15] reports a method for determining protein synthesis using ^2^H_2_O labelling *in vivo* and matrix-assisted laser desorption ionisation–time of flight mass spectrometry (MALDI-TOF MS) of rat serum proteins. We have used a similar approach to investigate the synthesis of eight proteins in four striated muscles, including the heart, diaphragm, extensor digitorum longus (EDL) and soleus.

Striated muscles are highly specialised tissues that exhibit a broad range of different phenotypic properties particularly with regard to speed of contraction and fatigue resistance [16]. The diversity of muscle phenotypes is largely due to differential expression of different isoforms of key contractile proteins, which concomitantly dictates the relative abundance of enzymes involved in substrate utilisation and energy production. In the past, we have reported the relative abundance of proteins in various rat striated muscles using proteomic techniques including 2DGE and MALDI-TOF MS e.g., [17,18,19] but there is currently a lack of equivalent data regarding synthesis rates of rat muscle proteins. In particular, we sought to investigate whether the rank order of synthesis rates for a selection of proteins is similar across different striated muscles or if enzymes that exist at a greater concentration in a particular muscle also exhibit a more rapid rate of renewal.

## 2. Materials and Methods 

Experimental procedures were conducted under the British Home Office Animals (Scientific Procedures) Act 1986 and were approved by the local ethical review committee. Male Wistar rats (352 ± 30 g body weight) were bred in-house in a conventional colony, housed in controlled conditions of 20 °C, 45% relative humidity, and a 12 h light (0600–1800 hours) and 12 h dark cycle, with water and food available *ad libitum*. 

Animals were assigned to four groups (*n* = 3, in each), including a control group and three groups that received deuterium oxide (^2^H_2_O) for either 4 days, 7 days or 14 days. Deuterium administration was initiated by an intraperitoneal injection of 10 μL.g 99% ^2^H_2_O-saline, and was maintained by administration of 5% (*v*/*v*) ^2^H_2_O in drinking water, which was refreshed daily. Animals were asphyxiated with a rising concentration of CO_2_ and killed by cervical dislocation. Samples of the heart (HRT) and diaphragm (DIA), and the entire extensor digitorium longus (EDL) and soleus (SOL) were isolated. Each muscle was cleaned of fat and connective tissue and then weighed before being frozen in liquid nitrogen and stored at −80 °C. 

Muscles were ground under liquid nitrogen and a portion (~100 mg) homogenised on ice in 10 volumes of 7 M urea, 2 M thiourea, 4% (*w*/*v*) CHAPS, 40 mM Tris pH 7.4 including phosphatase inhibitor and complete protease inhibitor cocktails (Roche, Indianapolis, USA). After centrifugation at 12,000× *g*, 4 °C for 45 min the supernatant was decanted and the protein concentration of a 5 μL aliquot measured using a modified “microtitre plate“ version of the Bradford assay (Sigma, Poole, Dorset, UK). 

Muscle homogenates were prepared for 2-dimensional gel electrophoresis (2DGE) as described previously [18]. An aliquot of each supernatant was precipitated in acetone and resuspended in 7 M urea, 2 M Thiourea, 2% (*w*/*v*) CHAPS, 20 mM dithiothreitol, 0.5% (*v*/*v*) ampholytes. Samples, containing 250 mg protein, were loaded on to 13 cm pH 3–11 nonlinear IPG strips (GE Healthcare, Chalfont St Giles, UK) and focused using an “active rehydration” and isoelectric focusing protocol comprising: 150 Vh at 30 V, 300 Vh at 60 V, 500 Vh at 500 V, 1000 Vh at 1000 V and 48 000 Vh at 8000 V; conducted on an IPGPhor II (GE Healthcare) at 20 °C, maximum 50 mA per strip. IPG strips were equilibrated in 50 mM Tris-HCl pH 8.8, containing 6 M urea, 30% (*v*/*v*) glycerol, 70 mM SDS and a trace of bromophenol blue. DTT (65 mM) was present as a reducing agent in the first equilibration and iodoacetamide (135 mM) in the second. Proteins were electrophoresed through 16 cm linear 12% polyacrylamide gels at 20 °C; at a constant current of 15 mA per gel for 30 min, then 30 mA per gel until the tracking dye reached the bottom edge of the gel. Gels were washed and stained with colloidal Coomassie blue (Bio-Safe; BioRad, Hercules, CA, USA) according to the manufacturer’s instructions.

Gel spots were cut and processed using an Xcise robot (Proteome Systems, North Ryde, Australia) directed by the gel analysis software. Gel plugs destained in three changes of 25 mM ammonium bicarbonate in 50% acetonitrile were dehydrated before being incubated with 35 µL of 1.25 mg/mL porcine trypsin (Promega, Madison, WI, USA) in 50 mM ammonium bicarbonate. Peptide solutions were de-salted and concentrated (Zip-tips; Millipore, Billercia, MA, USA) before being mixed with matrix (5 mg/mL α-cyano-4-hydroxcinnamic acid in 50:50 acetonitrile and 0.1% trifluoroacetic acid) and spotted on to 384-well stainless steel target plates. A calibration mix (Laserbio Labs, Sophia Antipolis, France) consisting of angiotensin II (*m/z* 1046.2), angiotensin I (*m*/*z* 1296.5), neurotensin (*m*/*z* 1672.9), ACTH fragment {1–17} (*m*/*z* 2093.5) and ACTH fragment {18–39} (*m*/*z* 2465.19) was mixed 1:1 with matrix solution and spotted (0.5 µL) between every four sample-wells. Peptide mass spectra were recorded using a matrix-assisted laser desorption ionisation tandem time of flight (MALDI-TOF/TOF) mass spectrometer (Axima TOF^2^; Shimadzu Biotech, Manchester, UK) in positive reflectron mode over a mass/charge (*m*/*z*) range of 900–3000. Data were smoothed (Gaussian, 2 chan peak width), baseline subtracted (100 chan peak width) and an adaptive (8.0×) threshold applied. Peptide mass lists (restricted to 20 peptides over 900–3000 *m/z*) were produced using the peak selection tool of the instrument's Launchpad software (Version 2.8.4) and searched against the Swiss-Prot database restricted to “Rattus” using the online MASCOT (www.matrixscience.com) server. The enzyme specificity was set as trypsin allowing one missed cleavage, carbamidomethyl modification of cysteine (fixed), oxidation of methionine (variable) and an *m/z* error of ±0.3 Da. 

The amount of newly synthesised protein was calculated in deuterium labelled samples using mass isotopomer distribution analysis [20]. Peptides were resolved to mass isotopomer envelopes. The pattern of mass isotopomers from deuterium-enriched samples was used to calculate the molar percent enrichment (MPE) of deuterium (^2^H) in the precursor pool (p) as well as the fraction of newly synthesised protein. Essentially, the enrichment of “heavy“ isotopomers (e.g., m_2_/m_1_) provides information on the level of ^2^H in the precursor pool, whereas the incorporation of ^2^H “heavy” isotopes in to newly synthesised protein causes the relative abundance of the monoisotopic peak (m_0_) to decline. Raw mass spectra were exported in mzXML format and mMass software (Version 5.5.0, http://www.mmass.org) was used to measure the intensities of mass isotopomers (m_0_, m_1_, m_2_) of peptides of interest, and to calculate the elemental composition of each peptide. The number (*N*) of exchangeable H–C bonds in each peptide was estimated from [21] and subsequent data handling was performed in R (www.R-project.org). Mass isotopomer intensities were converted to relative abundance of each mass isotopomer envelope and mass isotopomer distribution analysis (MIDA) tables were created using multinomial distribution, reported in detail in [20]. When the number of exchangeable H–C bonds and the elemental composition of the peptide are known, the level of precursor enrichment can be calculated from the ratio of enrichment between “heavy” isotopomers (*i.e.,* em_2_/em_1_). When the level of precursor enrichment is known the rise in the m_1_/m_0_ ratio due to ^2^H incorporation can be used to calculate fractional synthesis. Fractional synthesis was calculated from the slope of the m_1_/m_0_ regression line created from modelled data of each peptide based on its natural distribution of C, H, N, O elements, the number of exchangeable H–C bonds and the measured enrichment of ^2^H in the precursor pool.

Statistical analyses were conducted in Prism v6.02/6.0c (GraphPad, La Jolla, CA, USA); nonlinear one-phase association was used to estimate the rate constant *k* and half-life of each protein based on the amount of newly synthesised protein measured after 4 days, 7 days and 14 days of deuterium administration. The coefficient of determination (R^2^ value) was used as a measure of the goodness of the fitted non-linear regression and one-way ANOVA was used to investigate significant differences of individual protein synthesis rates across muscle tissues. The level of statistical significance was set at *p* < 0.05.

## 3. Results

Protein synthesis *in vivo* was investigated in the diaphragm, heart, EDL and soleus of rats administered ^2^H_2_O for either 4 days, 7 days or 14 days. Control (0-day) muscles were harvested from animals that did not receive deuterium. There was no significant difference in the wet weight of muscles harvested at each of the time points investigated. 

**Figure 1:**
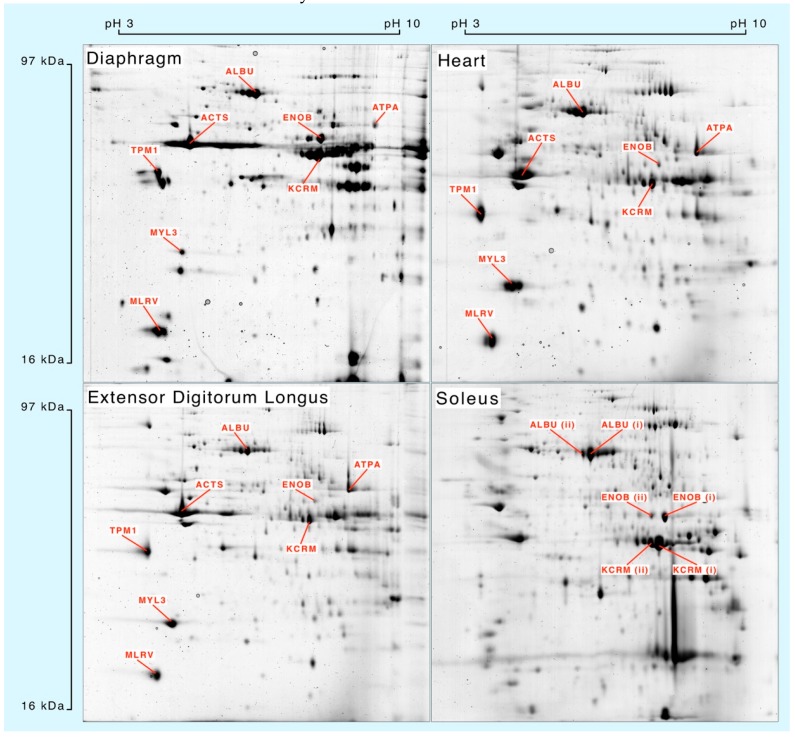
Separation of muscle proteins by 2-dimensional gel electrophoresis.

Muscle homogenates were separated by 2DGE (Figure 1) and eight spots matched across each muscle were processed for mass spectrometry. Peptide mass fingerprinting (Table 1) was used to confirm the identification of albumin (ALBU), actin (ACTS), β-enolase (ENOB), muscle creatine kinase (KCRM), ATP synthase-α (ATPA), tropomyosin α-1 (TPM1), essential myosin light chain (MYL3) and regulatory myosin light chain (MLRV). Soleus muscle samples were used to investigate synthesis of different species of ALBU, ENOB and KCRM that each exhibited similar relative molecular mass but different isoelectric points.

**Figure 2:**
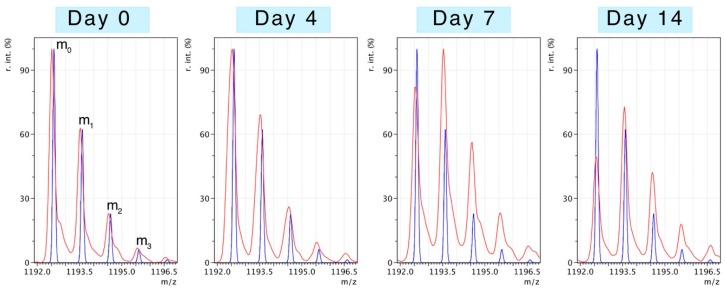
Mass spectrometry of deuterium-labelled peptides. Peptides were resolved as series of mass isotopomers (m_0_, m_1_, m_2_, ...) using matrix-assisted laser desorption ionisation mass spectrometry (MALDI-MS). Experimental mass spectra (red traces) from peptide DGFIDKNDLR (residues 41–50 of slow/ventricular myosin regulatory light chain; MLRV) are displayed from samples taken after 0, 4, 7 or 14 days of deuterium oxide (^2^H_2_O) administration *in vivo*. The blue trace represents the distribution of mass isotopomers predicted from the elemental composition of the peptide and the natural abundances of ^12^C, ^1^H, ^14^N and ^16^O (mMass software). The m_0_ (monoisotopic) peak is composed entirely of primary isotopes (*i.e.*, ^12^C, ^1^H,^14^N and ^16^O), whereas m_1_, m_2_, m_3_ contain either 1, 2 or 3 “heavy“ isotopes (e.g., ^13^C, ^2^H, *etc*.).

Peptides selected for mass isotopomer distribution analysis (MIDA) were of relatively high intensity and signal:noise ratio, rich in alanine and were specific to the identified protein. With the exception of ATPA (three peptides), five peptides were analysed for each protein. Table 1 provides details of the sequence, monoisotopic peak and elemental composition of the selected peptides. The number of exchangeable H–C bonds was estimated from published data on tritium incorporation in to amino acids *in vivo* [21]. Figure 2 illustrates changes to the pattern of peptide mass isotopomers due to the incorporation of ^2^H during protein synthesis *in vivo*. Peptides were resolved to mass isotopomer envelopes consisting of the monoisotopic (m_0_) peak, and “heavy” isotopomer peaks (m_1_, m_2_, m_3_). The ratio between “heavy“ isotopomers (e.g., m_2_/m_1_) provides information on the enrichment of ^2^H in the precursor pool. The incorporation of ^2^H “heavy” isotopes in to newly synthesised protein causes the relative abundance of the monoisotopic peak (m_0_) to decline and the ratio of m_1_/m_0_ to increase. When the level of precursor enrichment is known the rise in the m_1_/m_0_ ratio due to ^2^H incorporation can be used to calculate synthesis.

Whole muscle homogenates resolved by 2-dimensional gel electrophoresis and stained with colloidal Coomassie blue. Protein identities were confirmed by peptide mass fingerprinting (Table 1) of in-gel digests analysed by matrix-assisted laser desorption ionisation mass spectrometry. 

Fractional synthesis was calculated from the slope of the m_1_/m_0_ regression line created from modelled data, which is equivalent to the use of MIDA tables reported previously [20]. Briefly, regression was based on the natural distribution of C, H, N, O elements, the number (*N*) of exchangeable H–C bonds (H–D in Table 1) and the molar percent enrichment (MPE) of ^2^H in the precursor pool (p). Table 2 provides an example of data modelled from peptide DGFIDKNDLR displayed in Figure 2. To illustrate the method in this example the molar fraction of mass isotopomers was calculated using multinomial distribution based on the natural abundance of ^13^C only and the rate of protein degradation was assumed to be constant. Two different scenarios have been modelled. In Table 2a, differences in the fraction (%) of newly synthesised protein can be seen to alter the ratio between “heavy“ and “light“ (e.g., m_1_/m_0_) isotopomers while the pattern (ratio) of enrichment between “heavy“ isotopomers (*i.e.,* em_2_/em_1_) remains constant. Conversely, in Table 2b, enrichment of ^2^H in the precursor pool is varied between 0 and 2.5% while synthesis is held constant (100%). Under these conditions the pattern of enrichment of “heavy“ isotopomers (*i.e.*, em_2_/em_1_) changes in accordance with the level of precursor enrichment. This ability to distinguish between the effects of protein synthesis and precursor enrichment is fundamental to the use of MIDA. 

MPE of the precursor pool was calculated from the enrichment of the m_2_/m_1_ isotopomers of a selection of the best quality peptides (*n* = ~80). The average MPE from all peptides of ^2^H_2_O treated animals was 2.14% ± 0.2% and did not significantly differ between animals or across the time points (4 days, 7 days, 14 days) investigated. This is consistent with previous investigations, demonstrating rapid (<30 min in rats) equilibration of deuterium oxide in the body water compartment [13].

**Figure 3:**
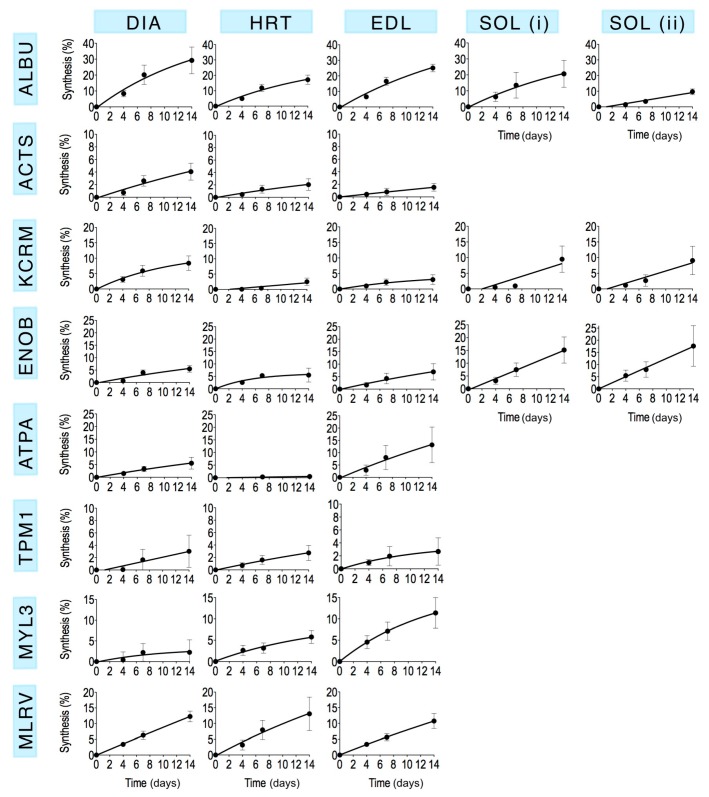
Time course of newly synthesised protein *in vivo*. The incorporation of deuterium *in vivo* was used to measure the synthesis of new protein using mass isotopomer distribution analysis. Data are presented as the average (Mean ± SEM) percentage of newly synthesised protein measured from 3–5 peptides for each protein, replicated in *n* = 3 biological samples. Data are fitted using a non-linear first-order equation. The range of the y-axis is consistent across muscles for each protein (*i.e.*, by row) by differs between proteins (*i.e.*, by column).

Mass spectra were collected from each of the selected gel spots (Figure 1) in each striated muscle harvested from control animals and rats that received ^2^H_2_O for either 4 days, 7 days or 14 days (*n* = 3 in each group). The fraction of newly synthesised protein after 4, 7 and 14 days calculated by MIDA was fitted using nonlinear first-order regression (Figure 3) to estimate the rate constant (*k*) of protein synthesis and the half-time of protein renewal (Table 3) assuming no change in protein abundance.

**Figure 4:**
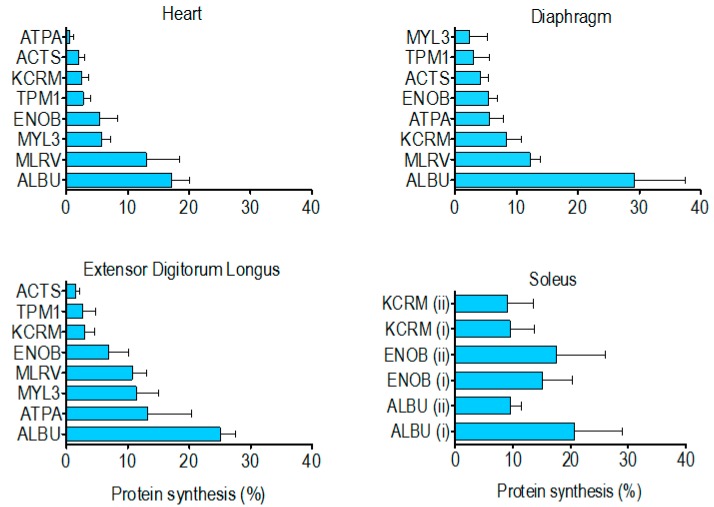
Rank order of newly synthesised protein within each striated muscle. Data represent percentage (mean ± SD) of new protein synthesised after 14 days, presented in relative rank order within the heart, diaphragm, extensor digitorium longus and soleus.

The percentage of newly synthesised protein in rat striated muscles after 14 days of ^2^H_2_O administration (Table 4) spanned from 0.5% ± 0.6% (heart ATP synthase alpha) to 29.2% ± 8.4% (diaphragm albumin). In each muscle investigated the synthesis of albumin was significantly (*p* < 0.05) greater than that of the other muscle proteins analysed. With the exception of albumin, the rank order of synthesis (Figure 4) was different across each of the muscles investigated. Moreover, in the soleus muscle, the synthesis of ALBU spot (i) was significantly (*p* < 0.05) greater than ALBU spot (ii), which provides evidence of a functional difference between these protein species.

## 4. Discussion

We report novel data regarding the synthesis of eight proteins in four striated muscles of the rat using deuterium oxide labelling *in vivo*, peptide mass spectrometry and mass isotopomer distribution analysis (MIDA; [20]). To the best of our knowledge, this is the first work to report the synthesis of individual proteins across a range of different rat striated muscles. One of our major findings was that the amount of protein synthesis measured here in free-living animals across a number of time points during a 14-day period was generally less than reported in previous studies that used short-term (e.g., 30 min) infusion of an amino acid tracer.

In the current work the synthesis of albumin (~23% after 14 days) in each of the muscles investigated was significantly greater than the range (0.5%–15.2% after 14 days) exhibited by the other muscle proteins. Albumin is a prominent feature of the skeletal muscle proteome [22] but expression of the albumin gene is low in muscle [23]. Therefore, our findings support the conclusion that albumin extracted from muscle is primarily synthesised in the liver, which typically has a more rapid turnover of proteins than striated muscle [24]. After removal of the myofibrillar components, albumin is one of the most abundant proteins in skeletal muscle [22,25]. Therefore, our data have consequences for the interpretation of previously reported synthesis measurements of mixed proteins from myofibrillar, sarcoplasmic and mitochondrial fractions e.g., [26]. The high abundance of albumin in the sarcoplasmic fraction of muscle is likely to have artificially raised the average synthesis rate reported for this fraction and may also have masked biologically important changes in the synthesis of less abundant muscle proteins in response to experimental interventions.

Skeletal muscle albumin is primarily localised to the interstitium [27] and is thought to aid transport of fatty acids in to muscle. The total abundance of albumin in fast-twitch skeletal muscle increases during transformation induced by chronic low frequency stimulation [28]. Similarly, intensity-controlled endurance training also increases the abundance of a specific species of albumin in the fast-twitch plantaris muscle of rats [18]. In the current work we investigated the synthesis of different species of three proteins in the soleus muscle (Figure 1), including albumin. The predominant albumin species {ALBU (i)} had a relatively high level of synthesis (20.6% ± 8.4% after 14 days), consistent with our findings in the other striated muscles (Table 4). In contrast, synthesis of the more acidic albumin species {ALBU (ii)} was significantly (*p* < 0.05) less (9.6% ± 1.8% after 14 days), and it is this species {ALBU (ii)} that is specifically increased in rat plantaris muscle in response to endurance exercise training [18]. The difference in isoelectric point between the ABLU (i) and ALBU (ii) species is likely to be due to a post-translational modification, and we show this modification is able to affect the turnover of the albumin protein. The modification to albumin may occur directly within the liver or secondarily in the periphery where the shift toward a more acidic isoelectric point might be associated with lesser uptake/residency in the muscle interstitium. Alternatively, because albumin mRNA can be detected (albeit at low levels) in skeletal muscle [23] the relatively acidic species of albumin {ALBU (ii)} might represent protein that was synthesised and modified in the muscle rather than the liver. In the future, inclusion of hepatic tissue alongside the collection of various striated muscles from endurance-trained or chronically stimulated animals might resolve some of these unanswered questions.

Contrary to our findings for species of albumin in the soleus, there was no difference in synthesis between the two species of either beta-enolase or creatine kinase (Figure 4). This indicates the post-translational modifications underlying the charge differences in these proteins did not alter their rate of synthesis. Nonetheless, further investigation encompassing all species from a greater number of proteins is needed to determine whether this is generally true for the majority of muscle proteins. Although 2DGE is relatively laborious and has limited scalability, we believe such data will be important to the interpretation of findings from high-throughput tandem mass spectrometry (e.g., LC-MS/MS) analyses of protein digests, which afford greater proteome coverage [25] but cannot distinguish between the different species of each protein. Wider analysis is also required to build a more complete picture of differences in the synthesis of proteins across different muscles. Our initial finding is that the rank order of renewal of proteins is different in the myocardium, mixed-fibre diaphragm, slow-twitch soleus and fast-twitch EDL (Figure 4). Of the two proteins (beta-enolase and creatine kinase) investigated in the soleus and EDL, the amount of protein synthesised after 14 d was greater (*NS*) in the soleus (Table 4), which supports previous data [5,29] from protein mixtures, supporting the hypothesis that slow-twitch postural muscles have a higher rate of turnover than less frequently activated fast twitch muscles such as EDL. However, on the whole, we found no simple relationship between myofibre phenotype/muscle activity pattern and the synthesis of individual proteins. Of the proteins investigated, the synthesis of tropomyosin (~3% after 14 days) was the most consistent across the different striated muscles whereas ATP synthase-α exhibited more than a 20-fold difference between the heart (0.5% after 14 days) and EDL (13% after 14 days). This finding is in accordance with earlier work [6] reporting that synthesis of the mitochondrial protein, cytochrome c, is greater in fast- than slow-twitch muscle.

As anticipated, the amount of protein synthesised during a 14-day period was less than predicted from extrapolation of short-term synthesis measurements. For example, Jaleel *et al*. [9] reports the synthesis of TPM1 is 8%/day based on stable isotope incorporation after short-term (~20 min) infusion of ring-[^13^C_6_] phenylalanine *in vivo*. In the current work, the rate constant for TPM1 was 2.7%/day (Table 3). Because incorporation of label from the precursor pool in to a newly synthesised protein exhibits non-linear first-order kinetics, this discrepancy is most likely due to linear extrapolation of data recorded after 20 min of infusion in [9]. It is also important to recognise that spots excised from 2DGE often contain multiple proteins [30]. Therefore, GC-MS analysis of amino acids hydrolysed from 2DGE spots (e.g., Jaleel *et al*. [9]) could include contamination from proteins that share the same relative mass and isoelectric point as the protein of interest. Arguably, the level of contamination may be small but is nonetheless an unknown variable that is able influence the results. In comparison, our current approach offers greater selectivity because both precursor enrichment and protein synthesis were quantified from protein-specific peptides, whereas unidentified peptides (*i.e.,* potential contaminants) were ignored.

Currently few data exist using comparable proteomic analysis of deuterium-labelled muscle proteins. Kasumov *et al*. [31] reports synthesis rates for 28 proteins in intermyofibrillar and subsarcolemmal mitochondria of the rat heart using ^2^H_2_O labeling and high-resolution peptide mass spectrometry. Protein synthesis was greater in the subsarcolemmal mitochondrial population, therefore, sub-cellular localisation is a further parameter that needs to be considered when investigating the synthesis of individual proteins. Kasumov *et al*. [31] reports the half-life of cardiac ATPA is approximately 27–30 days which is equivalent to the values estimated here in the diaphragm and EDL (Table 2). More recently, ^2^H_2_O labelling *in vivo* has been used to investigate synthesis in the triceps of ovariectomised rats exposed to a selective androgen receptor modulator or vehicle control [32]. Rats were administered deuterium oxide for a period of 7 days prior to muscle harvesting and peptide mass spectrometry was used to calculate the synthesis of individual proteins. Table 5 provides a comparison between our 7-day data and equivalent data reported in Table 2 of Shankaran *et al*. [32]. In most cases, we report less protein synthesis in the hindlimb muscles than Shankaran *et al.* report in the triceps. The exception is regulatory myosin light chain, which was synthesised similarly in each of the striated muscles investigated.

An advantage of deuterium oxide labelling compared to infusion of amino acid labelled tracers is that experiments can be conducted over longer durations and therefore provide a better estimation of the rate of protein synthesis. We used a minimum number of time points over the duration of the experiment to fit nonlinear regression curves to our data. However, incorporation of deuterium (*i.e.,* protein synthesis) did not reach a plateau for any of the proteins investigated during the 14-d period. Therefore, we report estimated rate constants of protein synthesis (Table 3) of each investigated protein and stress that it may be inappropriate to extrapolate values to estimate half-time for protein renewal or time for complete turnover of the protein pool. Instead, in Table 4, we report the fraction of newly synthesised protein after 14 days to enable a more robust comparison of the synthesis of individual proteins across the different striated muscles. Based on our current findings, the duration of future experiments should extended >3 weeks in order to capture a more accurate reflection of the individual protein synthesis rates within rat striated muscles.

## 5. Conclusions 

Our current work demonstrates that the synthesis of individual proteins differs not only within a muscle, but also the rank order of which protein exhibits the greatest synthesis is different, depending on the muscle investigated. We believe this work raises many exciting opportunities for further investigation. Not least, it will be interesting to discover how different muscle sub-fractions respond to muscle adaptation and if the rank order of synthesis in muscle is altered in response to important physiological and patho-physiological perturbations.

## Figures and Tables

**Table 1:** Protein identification by peptide mass fingerprinting.

Protein	Score	Coverage	Peptide Sequence	*m*/*z*	CHNO	H–D
ALBU	170	27%	CPYEEHIK	1075	C45H67N11O14	17
			FKDLGEQHFK	1248	C58H85N15O16	13
			FPNAEFAEITK	1266	C59H87N13O18	21
			LGEYGFQNAVLVR	1465	C67H104N18O19	18
			DVFLGTFLYEYSR	1609	C77H108N16O22	17
ACTS	86	28%	AGFAGDDAPR	976	C41H61N13O15	25
			GYSFVTTAER	1130	C50H75N13O17	17
			AVFPSIVGRPR	1198	C55H91N17O13	23
			QEYDEAGPSIVHR	1500	C64H97N19O23	29
			SYELPDGQVITIGNER	1791	C77H123N21O28	26
KCRM	117	30%	FEEILTR	907	C42H66N10O13	14
			GYTLPPHCSR	1187	C49H75N15O14	19
			DLFDPIIQDR	1231	C55H86N14O18	15
			SFLVWVNEEDHLR	1643	C75H110N20O22	21
			GTGGVDTAAVGAVFDISNADR	1992	C83H113N25O32	39
ENOB	124	39%	IGAEVYHHLK	1166	C54H83N15O14	19
			VVIGMDVAASEFYR	1556	C70H109N17O21	26
			VVIGMDVAASEFYR	1572	C70H109N17O21	26
			AAVPSGASTGIYEALELR	1804	C79H129N21O27	42
			LAMQEFMILPVGASSFK	1901	C86H137N19O23	26
ATPA	137	34%	AVDSLVPIGR	1026	C45H79N13O14	19
			GIRPAINVGLSVSR	1438	C62H111N21O18	31
			TGAIVDVPVGDELLGR	1611	C70H119N19O24	28
TPM1	163	38%	HIAEDADR	926	C37H59N13O15	23
			LDKENALDR	1073	C44H76N14O17	17
			LVIIESDLER	1186	C52H91N13O18	19
			KATDAEADVASLNR	1460	C59H101N19O24	31
			SIDDLEDELYAQK	1538	C59H101N19O24	23
MYL3	91	44%	HVLATLGER	995	C43H74N14O13	18
			EAFLLFDR	1010	C48H71N11O13	15
			DQGGYEDFVEGLR	1484	C64H93N17O24	23
			DTGTYEDFVEGLR	1501	C65H96N16O25	21
			NKDTGTYEDFVEGLR	1744	C75H114N20O28	22
MLRV	119	63%	VFDPEGKGSLK	1176	C53H85N13O17	17
			DGFIDKNDKR	1192	C51H81N15O18	13
			EAFTIMDQNR	1240	C51H81N15O18	15
			NLVHIITHGEEKD	1504	C65H105N19O22	21
			LKGADPEETILNAFK	1645	C74H120N18O24	26

Albumin (ALBU), skeletal muscle alpha actin (ACTS), skeletal muscle creatine kinase (KCRM), beta-enolase (ENOB), ATP synthase alpha (ATPA), tropomyosin alpha-1 (TPM1), myosin essential light chain 3 (MYL3), myosin regulatory light chain slow/ventricular (MLRV). Score (MOWSE) and coverage (percent sequence coverage) reported from peptide mass fingerprinting and mascot searches against the Rattus Swiss-Prot database. Monoisotopic peak (*m*/*z*), elemental composition (CHNO) and number of exchangeable H–C bonds (H–D) are reported for each peptide.

**Table:** (**a**)

Synthesis (%)	m0	m1	m2	m1/m0	m2/m1	em0	em1	em2	em1/em0	em2/em1
0	0.5797	0.3288	0.0914	0.5672	0.2781	--	--	--	--	--
25	0.5440	0.3450	0.1110	0.6341	0.3219	−0.0357	0.0161	0.0196	−0.4512	1.2162
50	0.5082	0.3611	0.1307	0.7105	0.3619	−0.0715	0.0323	0.0392	−0.4512	1.2162
75	0.4725	0.3772	0.1503	0.7983	0.3984	−0.1072	0.0484	0.0588	−0.4512	1.2162
100	0.4368	0.3933	0.1699	0.9006	0.4319	−0.1430	0.0645	0.0784	−0.4512	1.2162

**Table:** (**b**)

Precursor (%)	m0	m1	m2	m1/m0	m2/m1	em0	em1	em2	em1/em0	em2/em1
0	0.5797	0.3288	0.0914	0.5672	0.2781	--	--	--	--	--
0.5	0.5472	0.3461	0.1067	0.6326	0.3082	−0.0325	0.0173	0.0152	−0.5315	0.8816
1.0	0.5168	0.3610	0.1223	0.6986	0.3387	−0.0630	0.0321	0.0308	−0.5105	0.9587
1.5	0.4883	0.3737	0.1380	0.7652	0.3694	−0.0914	0.0448	0.0466	−0.4902	1.0399
2.0	0.4617	0.3844	0.1539	0.8325	0.4005	−0.1180	0.0555	0.0625	−0.4705	1.1256
2.5	0.4368	0.3933	0.1699	0.9006	0.4319	−0.1430	0.0645	0.0784	−0.4512	1.2162

Predicted molar fraction of mass isotopomers (m0, m1, m2) at 0% synthesis (2a) or 0% precursor enrichment (2b) is subtracted from the molar fraction of mass isotopomers that include deuterium to give the enriched molar fraction of mass isotopomers (em0, em1, em2). In Table 2a, m1/m0 ratio is linearly associated with the fraction of newly synthesised protein. In Table 2b, the em2/em1 ratio is linearly associated with the level of precursor enrichment; the slope of these relationships is determined by the number of exchangeable C-H sites and the intercept is principally determined by the number of C atoms in the peptide. For example, the straight line equation of em2/em1 for peptide DGFIDKNDLR (C-H = 13, C = 51) is y = 0.1672x + 0.7936.

**Table 3:** Half-time and rate constants (*k*) of protein synthesis in rat striated muscles calculated from 14 days ^2^H_2_O administration *in vivo*.

Protein	Heart	Diaphragm	EDL	Soleus
Albumin (ALBU)	11.0 (*k* = 0.063)	11.0 (*k* = 0.063)	14.4 (*k* = 0.048)	13.3 (*k* = 0.052)
Actin (ACTS)	20.2 (*k* = 0.034)	28.7 (*k* = 0.024)	87.8 (*k* = 0.008)	-
Creatine kinase (KCRM)	NA	7.8 (*k* = 0.089)	8.4 (*k* = 0.083)	NA
Beta enolase (ENOB)	NA	21.1 (*k* = 0.033)	24.2 (*k* = 0.029)	NA
ATP synthase α (ATPA)	NA	25.2 (*k* = 0.028)	28.9 (*k* = 0.024)	-
Tropomyosin (TPM1)	28.5 (*k* = 0.024)	NA	7.4 (*k* = 0.094)	-
Myosin essential light chain (MLY3)	10.8 (*k* = 0.064)	6.5 (*k* = 0.107)	9.3 (*k* = 0.074)	-
Myosin regulatory light chain (MLRV)	25.3 (*k* = 0.027)	NA	47.8 (*k* = 0.016)	-

Half-time in days and rate constant (*k*) of protein synthesis calculated from nonlinear regression of the change in newly synthesised protein measured at 4 days, 7 days and 14 days using mass isotopomer distribution analysis of deuterium-labelled muscles. NA = data not available due to inadequate data fitting.

**Table 4:** Percentage of newly synthesised protein in rat striated muscles after 14 days ^2^H_2_O administration *in vivo*.

Protein	Heart	Diaphragm	EDL	Soleus
Albumin (ALBU)	17.2 ± 3.0 *	29.2 ± 8.4 *	25.1 ± 2.5 *	20.6 ± 8.4 *
Actin (ACTS)	2.0 ± 0.9	4.1 ± 1.3	1.5 ± 0.6	-
Creatine kinase (KCRM)	2.5 ± 1.2	8.4 ± 2.3	3.0 ± 1.5	9.5 ± 4.2
Beta enolase (ENOB)	5.5 ± 2.8	5.5 ± 1.4	6.9 ± 3.2	15.2 ± 5.1
ATP synthase α (ATPA)	0.5 ± 0.6	5.6 ± 2.3	13.2 ± 7.2	-
Tropomyosin (TPM1)	2.7 ± 1.2	3.0 ± 2.6	2.7 ± 2.1	-
Myosin essential light chain (MLY3)	5.8 ± 1.5	2.2 ± 3.0	11.4 ± 3.6	-
Myosin regulatory light chain (MLRV)	13.1 ± 5.3	12.2 ± 1.7	10.7 ± 2.3	-

Percentage of newly synthesised protein measured after 14 days deuterium oxide labelling *in vivo.* * *p* < 0.05, significantly different from other Proteins. All data are presented as Mean ± SEM of *n* *= 3–*5 peptides from each protein measured in *n* = 3 animals.

**Table 5:** Percentage of newly synthesised protein in rat striated muscles after seven days D2O administration *in vivo*.

Protein	Heart	Diaphragm	EDL	Soleus	Triceps ^†^
Albumin (ALBU)	11.9 ± 2.4	20.2 ± 6.1	16.6 ± 2.7	13.6 ± 0.8	-
Actin (ACTS)	1.3 ± 0.6	2.6 ± 0.8	0.8 ± 0.5	-	7.0 ± 0.3
Creatine kinase (KCRM)	0.4 ± 0.3	5.9 ± 1.7	2.2 ± 1.0	1.0 ± 0.3	13.0 ± 0.4
Beta enolase (ENOB)	5.3 ± 0.3	4.0 ± 1.0	4.2 ± 1.2	7.5 ± 2.6	13.7 ± 0.3
ATP synthase α (ATPA)	0.3 ± 0.2	3.3 ± 1.0	8.1 ± 4.8	-	30.0 ± 1.2
Tropomyosin (TPM1)	1.6 ± 0.7	1.7 ± 1.6	2.0 ± 1.4	-	14.5 ± 0.4
Myosin essential light chain (MLY3)	1.2 ± 1.1	2.2 ± 2.1	7.2 ± 2.1	-	13.4 ± 1.6
Myosin regulatory light chain (MLRV)	7.9 ± 3.0	6.3 ± 1.3	5.6 ± 1.1	-	9.6 ± 0.3

^†^ From Table 2 of Shankaran *et al.* 2015 [32].

## Abbreviations

The following abbreviations are used in this manuscript:

MPE: Molar percent enrichment
GC-MS: Gas chromatography-mass spectrometry
EDL: Extensor digitorium longus
HRT: Heart
DIA: Diaphragm
SOL: Soleus
2DGE: 2 dimension gel electrophoresis
^2^H_2_O or D_2_O: Deuterium oxide
MALDI-TOF: Matrix assisted laser desorption ionization-time of flight
MS: Mass spectrometry
ALBU: Albumin
ACTS: Actin
ENOB: β-enolase
KCRM: Muscle creatine kinase
ATPA: ATP synthase-α
TPM1: Tropomyosin α-1
MYL3: Essential myosin light chain
MLRV: Regulatory myosin light chain
MIDA: Mass isotopomer distribution analysis
m_0_: Monoisotopic peak
LC-MS: Liquid chromatography-mass spectrometry
